# An Evaluation of Safety Training for a Diverse Disaster Response Workforce: The Case of the Deepwater Horizon Oil Spill

**DOI:** 10.3390/ejihpe11040116

**Published:** 2021-12-13

**Authors:** Sue Ann Sarpy, Michael J. Burke

**Affiliations:** 1 Sarpy and Associates, LLC, Charlottesville, VA 22903, USA; 2 A.B. Freeman School of Business, Tulane University, New Orleans, LA 70118, USA; mburke1@tulane.edu

**Keywords:** safety training, safety performance, disaster response, immigrant workers, minority workers

## Abstract

(1) Background: In this case study, we examined the safety-training-related experiences of individuals from six racial-ethnic groups (Asians (Vietnamese), Blacks, Hispanics, Isleños, Native Americans, and Whites) involved in the cleanup of the Deepwater Horizon oil spill. (2) Methods: We assessed, via a survey, 495 disaster response trainees’ reactions to the design and delivery of training, learning, safety performance, and injury and illness experience. (3) Results: Our results showed statistically significant racial-ethnic group differences with respect to reactions to training, components of learning (i.e., cognitive, skill, and affective), and safety performance (i.e., use of personal protective equipment, engaging in safe work practices, communicating of safety information, and exercising employee rights and responsibilities). In general, Asians and Isleños group members had lower reactions to training, self-reported learning, and safety performance. Additionally, we found that the safety climate interacted with learning to positively affect safety performance. (4) Conclusions: We discuss the implications of our findings for improving the quality of safety training in relation to addressing language and literacy concerns, developing training that is useful and engaging for volunteer and other cleanup workers from the contaminated region, and promoting a positive safety climate to enhance training transfer.

## 1. Introduction

High profile disasters such as the Exxon Valdez oil spill, Hurricane Katrina, and Deepwater Horizon oil spill have forced U.S. public officials, agencies, and accrediting entities to scrutinize disaster planning. This close examination has focused heavily on the preparation of workers for handling disasters and cleaning up in the wake of disasters [1,2,3,4]. Recent events, such as the oil spill in Orange County, California, demonstrate this ongoing need [5]. Concerning worker preparation, several authors and agencies have identified safety and health training as a critical need, because many disaster response workers are often non-traditional responders [3,6]. That is, the latter individuals are often previously unemployed or volunteer workers who are also racial-ethnic minorities, immigrants, and indigenous (Native American) people inexperienced in working with hazardous materials [3,6].

To date, we have limited information on the effectiveness of safety training for non-traditional disaster responders. In particular, the literature on the effectiveness of safety training for disaster cleanup workers is largely comprised of narrative reports of “lessons learned” and summaries of observations of cleanup workers [3,4,6]. This point is notable, as over 15 years ago, the U.S. Executive Office of the President & United States [4] called for not only improvements in safety training for disaster cleanup workers but also emphasized that “… assessments of training and exercises should be based on clear and consistent performance measures (p. 74)”. Despite such a call, there is little rigorous scientific research, using standardized measures or concepts, to evaluate the effectiveness of safety training for disaster cleanup workers. This situation is compounded by the reality that many disaster cleanup workers in the U.S., as racial-ethnic minorities, immigrants, and Native Americans are known to encounter language and cultural barriers to the acquisition of safety knowledge and engagement in safe work behavior [7,8,9]. Notably, this point concerning barriers to the acquisition and use of safety knowledge holds for other minorities, immigrants, and indigenous peoples in safety-critical work in other countries [10,11].

To address this gap in our understanding of the effectiveness of safety training for disaster response workers, this investigation examines the safety-training-related experiences of disaster cleanup workers including racial-ethnic minorities, immigrants, and Native Americans who participated in the Deepwater Horizon oil spill cleanup. The Deepwater Horizon oil spill that began on 20 April 2010 in the Gulf of Mexico on the BP-operated Macondo Prospect is considered the largest marine oil spill in the history of the petroleum industry. Importantly, the present case study and evaluation of the safety training for the Deepwater Horizon oil spill cleanup builds on both the general training evaluation literature and the general safety performance literature to examine the effectiveness of disaster workers’ safety training. In doing so, our study demonstrates how safety training professionals can apply a training evaluation framework to the assessment of disaster workers’ safety training and adapt a general model of safety performance for the assessment of training transfer for disaster cleanup workers. As such, our investigation is consistent with general calls for more rigor and the use of theoretical models in safety training evaluation research [12,13,14]. On a more specific level, our case analysis of the effectiveness of safety training in the wake of the Deepwater Horizon incident provides important information for improving safety training for disaster cleanup workers including racial-ethnic minorities, immigrants, and Native Americans in the Gulf of Mexico region of the U.S. Furthermore, while not an original intent of our case study, we will discuss possible broader implications of our evaluation effort for systematically assessing the training experiences of disaster cleanup workers beyond oil spills.

### 1.1. Deepwater Horizon Oil Spill and Disaster Worker Safety Training

On April 20, 2010, the BP Deepwater Horizon oilrig exploded in the Gulf of Mexico causing a massive oil release that directly affected four U.S. states: Louisiana, Alabama, Mississippi, and Florida. In response to the oil spill, the U.S. National Institute of Environmental Health Sciences (NIEHS) facilitated the presentation of health and safety training and development of training materials for over 147,000 cleanup workers (e.g., on-shore and off-shore volunteers, technical specialists) [15]. Approximately 47,000 trained workers participated in cleanup activities.

When the enormity of the training needs became clear under the coordinated federal response to the Deepwater Horizon incident, NIEHS’s Worker Education and Training Program (WETP) was asked to assist with worker safety and health training. A primary reason for the NIEHS WETP and its National Clearinghouse for Worker Safety and Health Training to join the training effort was that thousands of non-traditional responders (i.e., local fishermen and unemployed workers and volunteers) would need specialized curricula that would adequately prepare them for the cleanup tasks. In cooperation with the Occupational Safety and Health Administration (OSHA), NIEHS developed a training tool comprised of four modules that guided worker safety training, and an accompanying booklet serving as a resource document that workers could reference in the field during the response [15]. The modules included an introduction to oil spill cleanup, heat injury prevention, oil spill cleanup and health concerns, and other cleanup health and safety issues. Given that many cleanup workers were speakers of English as a second language or speakers of Vietnamese or Spanish, the National Clearinghouse for Worker Safety and Health Training translated this document into Spanish and Vietnamese.

Further, OSHA assigned 25 to 40 professionals solely to the oil spill response in order to ensure adequate worker preparation and protection. These professionals made over 4200 site visits, covering: training sessions, staging areas; decontamination, distribution, and deployment sites; and “Vessels of Opportunity” (displaced fishing vessels involved in defensive booming) [6]. While these efforts were laudable regarding checks on worker preparation and protection, NIEHS officials recognized the need for a more systematic evaluation of worker safety and health training during a national disaster response [3]. This recognition led to the conduct of a comprehensive evaluation of the effectiveness of the Deepwater Horizon oil spill safety and health training with an emphasis on assessing the training experiences of the diverse workforce that cleaned up the oil spill. In doing so, the evaluation effort was organized with respect to Kirkpatrick’s [16] framework for training program evaluation. Below, we provide more detail on Kirkpatrick’s framework including how we used this framework to pose key questions that guided the evaluation.

### 1.2. Evaluating Disaster Cleanup Worker Training: Reactions, Learning, Behavior and Outcomes

When evaluating safety and health training, a number of authors have advocated for the use of Kirkpatrick’s [16] four-level evaluation framework [11,17,18]. Kirkpatrick’s framework considers reactions of participants to the training program (Level 1); learning resulting from training (Level 2); behavior following training (Level 3); and outcomes associated with the training program (Level 4). Yet, as Asari and Leman [17] pointed out, rarely do training professionals evaluate safety training systematically relative to Kirkpatrick’s framework, with safety training researchers most often evaluating training only at the first level (i.e., reactions).

In the interest of comprehensiveness, our evaluation of Deepwater Horizon oil spill disaster worker training considered all levels within Kirkpatrick’s [16] training evaluation framework. Below, for each level in Kirkpatrick’s framework, we pose an exploratory research question. While our research questions are exploratory in nature, a general expectation was that racial/ethnic minorities with language and literacy issues, in comparison to those without such issues, would have lower reactions to training, acquire less knowledge, and have a lower level of safe work behavior.

First, we considered trainees’ reactions in terms of the degree to which they believed that training was designed and delivered to address their needs. In doing so, we examined whether disaster cleanup workers from different indigenous, racial-ethnic and immigrant groups had different training experiences. More specifically, we addressed the following question:

Research Question 1 (Reactions): To what extent is oil spill safety training designed and delivered to address the needs of disaster cleanup workers from different indigenous, racial-ethnic, and immigrant groups?

For Kirkpatrick’s [16] second level, learning, we distinguished between learning of cognitive, affective, and skill components as suggested by Kraiger, Ford, and Salas [19]. As pointed out by Peiro et al. [11], for some minority and migrant workers, these types of learning are essential, given the specificities of the safety and health risks they face. Notably, researchers often assess learning resulting from safety training by structured interviews, follow-up surveys, and focus groups (see Peiro et al. [11] for a systematic review in the construction industry). In line with this practice of self-assessment of learning, we empirically addressed the following question:

Research Question 2 (Learning): To what extent do oil spill cleanup workers from different indigenous, racial-ethnic, and migrant groups report affective, cognitive, and skill learning due to safety training?

In addition, to explore whether individual factors (e.g., language ability) or course-related design factors (e.g., training content, methods of delivery) promoted or adversely affected disaster cleanup workers’ learning, we qualitatively addressed the following question:

Research Question 3: What are the factors that facilitated or hindered learning for oil spill cleanup workers?

Kirkpatrick’s [16] third level, behavior, concerns the extent to which learning carries over to the work setting. Relatedly, translation of learning into the work context is a key feature of Baldwin and Ford’s [20] concept of “training transfer.” Concerning the transfer of safety training, Burke, Sarpy, Tesluk, and Smith-Crowe [21] developed a four-factor model of worker safety performance and demonstrated how it applied across numerous jobs and occupational fields. The behavior/performance factors comprising this model include using personal protective equipment, engaging in work practices to reduce risk, communicating safety and health information, and exercising employee rights and responsibilities. Notably, this four-factor model has served as the basis for measuring safety performance in numerous studies as well as the model for evaluating training transfer with respect to several regional and national safety and health training initiatives [18,22]. Importantly, of the latter initiatives, the four-factor safety performance model served as the guide for the U.S. Department of Energy’s efforts to evaluate the transfer of hazardous waste worker safety training for the tens of thousands of trainees in the wake of the Cold War [21]. Given the relevance of the four-factor safety performance model for disaster cleanup, we employed it in addressing the following question:

Research Question 4 (Behavior): To what extent do oil spill cleanup workers from different indigenous, racial-ethnic and immigrant groups transfer knowledge and skill via actions taken (i.e., engaging in safe work behavior) to the cleanup site?

Concerning training transfer, a number of studies have indicated that transfer of safety training can be affected by work context factors such as performance pressures, managerial support, and safety climate [23,24]. For instance, in a study of 23 work units from two petrochemical companies, found that work unit safety climate moderated the relationship between workers’ self-reports of safety skills and safety performance [23]. Given that situational factors may hinder as well as support training transfer, we qualitatively addressed the following question:

Research Question 5: What are the factors that facilitate or hinder training transfer among oil spill cleanup workers?

In addition, we quantitatively evaluated the expectation that the relationship between learning and safety performance would be moderated by safety climate, with the relationship posited to be the stronger in more positive safety climates. Given that oil spill cleanup workers worked in small groups or on individual vessels of opportunity, we were interested in knowing the extent to which safety climate perceptions pertaining to supervisory support for the transfer of training and the provision of personal protective equipment moderated the learning-safety performance relationship. This expectation is grounded in the body of research indicating that a positive safety climate enhances the effect of interventions designed to promote safe work behavior [22].

Finally, in line with Level 4 of Kirkpatrick’s [16] training evaluation model, we explored whether disaster cleanup workers experienced any accidents, injuries, or illnesses during cleanup activities. In doing so, we attempted to answer the following question with an emphasis on the outcomes for different indigenous, racial-ethnic, and immigrant groups:

Research Question 6: Do oil spill cleanup workers themselves or their co-workers experience any accidents, illnesses, and injuries?

## 2. Materials and Methods

### 2.1. Participants

As stated previously, approximately 47,000 trained workers participated in the cleanup activities. All workers (i.e., on-shore volunteers, offshore volunteers, technical specialists) who received safety training and/or who worked on oil spill cleanup were potential study participants. However, due to the inaccessibility of the safety training rosters, our safety training evaluation effort was based on a much smaller, convenience sample of individuals identified through NIEHS partners/community-based organizations (e.g., Boat People SOS, Bayou Interfaith Shared Community Organizing). These community-based organizations facilitated face-to-face administration of the evaluation survey to workers who remained in contact with the respective organizations. Of the approximately 600 workers who the community-based organizations asked to participate in this study, 495 provided usable survey responses. Among the responding workers, 74% were male (26% female). These respondents reported their racial-ethnic status as Black (27.6%), White (23%), Asian (Vietnamese, 22%), Isleños (19.5%), Hispanic (6.1%), and Native American (1.8%). Vietnamese and Isleños are, respectively, Vietnamese and Canary Island emigrants to the U.S. Gulf Coast region and their descendants. Native Americans, also known as American Indians, are the indigenous peoples of the United States.

### 2.2. Safety Training Intervention

Training was developed and delivered in modules, where the vast majority of workers participated in either multiple training modules or the more advanced hazardous waste cleanup course. The training courses focused on engaging in safe work practices, using personal protective equipment, engaging in decontamination, and understanding heat stress and other hazards associated with cleanup work [15]. A complementary booklet highlighting the general training concepts also was developed and translated into Spanish and Vietnamese [15]. While we describe the modules separately below, we considered the training as a singular intervention due to the participation of almost all workers in either multiple modules or the more advanced Hazardous Waste Operations and Emergency Response (HAZWOPER) training course. The percentage of workers taking each module is noted below.

Module 1 was the BP health and safety orientation training for workers in non-contaminated environments (i.e., no contact with hazardous materials; pre-oil landfall beach cleanup). Module 1 was presented in a 30–45 min session to volunteers for non-contaminated beach cleanup and workers who were involved with pre-cleaning of the beaches (i.e., to pick-up trash and debris). Thirty-four percent of our sample participated in Module 1.

Module 2 was a 45 min orientation to BP procedures and was geared toward contract employees who were conducting work on behalf of BP in the field. This module was presented to individuals whose work would not involve spill-contaminated materials (e.g., site health, safety, and environmental orientation). Thirty percent of our sample participated in Module 2.

There were two components to Module 3: offshore awareness training was delivered to workers who were involved in offshore cleanup activities, whereas the onshore awareness training was delivered to workers who were cleaning contaminated shoreline environments. Module 3 training lasted four hours and was directed at individuals who had no previous experience with cleanup activity. More specifically, the offshore training was designed for individuals involved in marine cleanup (i.e., captains or crewmen working on Vessels of Opportunity). These workers were involved in skimming and setting oil (containment) booms or controlled burns. Onshore training was particularly designed for workers who were picking up tar balls and other oil-contaminated debris on beaches and along shoreline (i.e., beach cleanup workers). For the offshore and onshore versions of Module 3, 45% and 44% of our sample respectively participated in these modules.

Module 4 was an expanded offshore cleanup training targeted at vessel workers, captains or crewmen working on Vessels of Opportunity. Twenty-one percent of our sample participated in this module. Finally, 40 h (HAZWOPER) training was given to supervisors of workers who had direct contact with contamination, as well as to on-shore and off-shore workers. The HAZWOPER course was a 5 day course and is part of ongoing worker training offered through NIEHS and OSHA. Approximately 58% of the workers in our sample took this course.

### 2.3. Safety Training Evaluation Survey and Scoring of Variables

A training evaluation survey was developed to assess training relative to the four levels discussed above. Along with demographic information, workers were asked to indicate all types of training that they attended, if certifications were received for those trainings, and where they performed cleanup activities. With respect to Level 1 (Reactions to Training) assessments, the survey prompted workers to rate their perceptions of the design and delivery of training. Given our focus on racial-ethnic differences, we examined response to three key items. These items concerned the degree to which training was presented according to worker needs (e.g., culture, language, education level), the ease of understanding training materials, and the adequacy of training for their oil spill cleanup activities: Workers rated the items on a 7-point scale with anchors ranging from Strongly Disagree to Strongly Agree. The items pertaining to workers’ reactions to the design and delivery of training are presented in the Appendix A. Additionally, a composite, average item score, was computed for Reactions to Training.

For Level 2 (Learning) assessments, workers were asked to rate their acquisition of knowledge, empowerment, and skill for performing cleanup activities. These assessments corresponded to the cognitive, affective, and skill components of learning. Workers rated an item pertaining to each component of learning (see items in Appendix A) on a 7-point scale, with anchors ranging from Strongly Disagree to Strongly Agree. A learning composite was computed as the average of the three learning items.

With respect to Level 3 (Behavior) assessments, the survey prompted workers to rate 19 items concerning the frequency to which that they demonstrated safe work behavior. The 19 items are presented in the Appendix A. Workers rated these items on a 7-point scale with anchors ranging from Never to Always. Notably, our item development was guided by the theoretically grounded general safety performance model and scale (GSS) [21]. In doing so, some original items of the GSS were modified, with other items related to oil spill cleanup being developed for the four dimensions: Using Personal Protective Equipment (5 items); Engaging in Work Practices to Reduce Risk (7 items); Communicating Safety and Health Information (4 items); and Exercising Employee Rights and Responsibilities (3 items). In addition, if workers did not perform any activity, the survey asked them to provide an explanation for why they did not engage in that activity. Given systematic missing data on items pertaining to Exercising Employee Rights and Responsibilities, we computed an overall Safety Performance score as an average item score excluding the Exercising Employee Rights and Responsibilities items. The Exercising Employee Rights and Responsibilities scale was examined separately.

Safety climate was measured with respect to two items developed to assess management’s commitment and support of worker safety [25]. More specifically, the items focused on management support for the transfer of training and the provision of appropriate personal protective equipment. These items are presented in the Appendix A and were rated on 7-point scales.

To gather Level 4 information, the survey asked workers if they or any of their co-workers had experienced an accident, injury, or illness during cleanup operations. These questions were posed as exploratory, open-ended questions to gather all types of information pertaining to safety and health outcomes associated with the oil spill cleanup.

### 2.4. Analyses

The sample size available for analyses involving reaction and learning data was 495, whereas the sample size available for analyses concerning safety performance was 386. The primary reason for this difference in sample sizes is that 75 trainees did not perform oil spill cleanup work (e.g., boat sat idle, not called in to conduct cleanup work), but did attend training and provide useful responses to reaction and learning assessments. This percentage of trainees who did not perform oil spill cleanup in our sample (i.e., 15%) is far less than the percentage in the population of trainees, where approximately 68% did not engage in oil spill cleanup.

When addressing Research Questions 1 (Reactions to Training), 2 (Learning), and 4 (Behavior), which concern the comparison of means for the six racial-ethnic groups, we addressed these questions with respect to overall assessments as well as in regard to relevant items or dimensions. Initially, we employed an ANOVA for an omnibus test of group differences. A power analysis for a power of 0.80, with a root mean square standardized effect of 0.20 at the 0.05 level of significance, indicated that a sample size of 294 was needed. Based on this power analysis, all ANOVAs had adequate statistical power for detecting a small effect across groups.

For all comparisons of means, when we observed a significant omnibus test, we conducted post-hoc tests for group differences with Tukey’s HSD (Honest Significant Difference) test. In addition, to evaluate practical differences between racial-ethnic groups on the overall assessments for reactions, learning, and behavior, we computed Cohen’s d-statistic for differences between subsets of racial-ethnic groups identified via the Tukey HSD tests [26]. Cohen’s d-statistic provides a standardized measure of the extent to which two groups (in our case subsets of racial-ethnic groups) differ. When addressing Research Questions 3, 5, and 6, we examined the content of worker responses to determine if common themes/incidents emerged or occurred.

We employed a regression analysis with centered predictor variables to examine the expectation that safety climate would interact with learning in the prediction of overall safety performance. That is, prior to conducting this regression analysis, the predictor variables, safety climate and learning, were centered and the product (interaction) term was computed by multiplying the two centered variables. A power analysis for a regression with power of 0.80, with three predictor variables at the 0.05 level of significance, indicated that the needed sample size for detecting a small multiple R-squared change of 0.05 was 167. As such, our regression analysis with 239 workers had adequate statistical power. Workers needed to respond to both safety climate items to be included in the regression analysis involving safety climate, which reduced the available sample size for that analysis. For all analyses, other than those involving safety climate and Exercising Employee Rights (where data were systematically missing), mean substitution was employed for sporadically missing data.

## 3. Results

We organize the presentation of results according to the research questions that we posed. Prior to presenting the findings, we present the descriptive statistics and correlations between the study variables in Table 1.

### Effectiveness of Disaster Cleanup Worker Training: Reactions, Learning, Behavior and Outcomes

**Figure 1:**
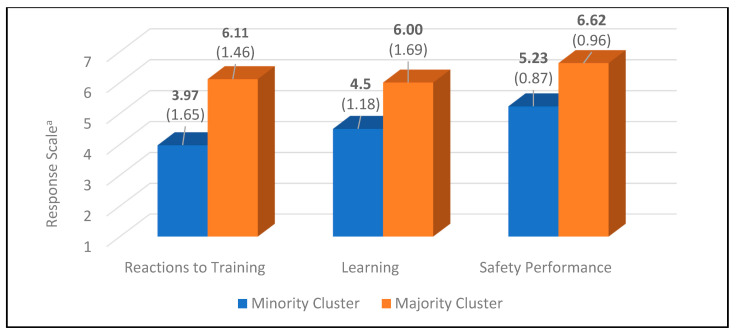
Mean differences (and standard deviations) in overall training outcomes for racial-ethnic group clusters. Note. For reactions to training: minority cluster (i.e., Asians–Isleños), majority cluster (Black–Hispanic–Native American–White). For learning: minority cluster (i.e., Asian–Isleños–Native American), majority cluster (Black–Hispanic–White). For safety performance: minority cluster (i.e., Isleños), majority cluster (Asian–Black–Hispanic–Native American–White). ^a^ The 7-point response scales for Reactions to Training and Learning ranged from strongly disagree to strongly agree. The 7-point response scale for Safety Performance ranged from never to always.

In answer to Research Question 1, “To what extent is safety training designed and delivered to address the needs of disaster cleanup workers from different indigenous, racial-ethnic, and immigrant groups?”, we found statistically significant group differences in assessments of the design and delivery of training. As reported in Table 2, while we observed differences in reactions to training relative to three subsets, Isleños and Asian trainees, in comparison to other group members (Black, Hispanic, Native American, and White trainees), generally had significantly lower reactions to training. The chart in Figure 1 presents mean differences between these two racial-ethnic group clusters on the overall training reactions measure. From a practical perspective, as shown in Table 3, the standardized differences between the Isleños–Asian cluster’s reactions to training in comparison to the other groups was 1.43. Notably, the d-statistic of 1.43 indicated that the mean of the grouping with the most positive reaction to training (i.e., Blacks, Hispanics, Native Americans, and Whites) was at approximately the 92nd percentile of the Isleños–Asian cluster. In general, this pattern of racial-ethnic group difference was also observed for the more specific assessment of training pertaining to meeting the needs of trainees, the usefulness of training materials, and the adequacy of training for Deepwater Horizon oil spill cleanup operations. The latter comparisons are presented in Table 4.

In addressing Research Question 2, “To what extent do disaster cleanup workers from different indigenous, racial-ethnic, and migrant groups report affective, cognitive, and skill learning due to of safety training?”, we observed significant group differences with respect to both an overall measure of learning and components of learning. The results pertaining to the overall measure of learning are presented in Table 2. As reported in Table 2, we observed differences in learning relative to three subsets. Although we observed differences in relation to three subsets, Black, Hispanic, and White trainees, in comparison to Asian, Isleños, and Native American trainees generally reported significantly greater acquisition of knowledge. The chart in Figure 1, presents mean differences between these two racial-ethnic group clusters on the overall learning measure. From a more practical perspective and as shown in Table 3, the d-statistic associated with the difference between these groupings was 1.01, which indicates that the mean of the grouping with greater knowledge acquisition (i.e., Blacks, Hispanics, and Whites) was approximately at the 84th percentile of the grouping with lessor knowledge gain (i.e., Asians, Isleños, and Native Americans). As shown in Table 5, this pattern of racial-ethnic group differences in knowledge acquisition was similarly observed for the components of learning. The primary differences being greater overlap in racial-ethnic groupings with respect to the affective (empowerment) component of learning.

In response to Research Question 3, “What are the factors that facilitated or hindered learning for disaster cleanup workers?”, we found several themes in workers’ open-ended replies that also clarified why the above subgroup differences in learning were reported. Notably, Isleños workers cited training design and delivery issues related to specific applications of training that did not promote learning. For example, they reported that the training they received often included examples of spill cleanup that were not applicable to the Deepwater Horizon oil spill cleanup effort (e.g., cleanup of oil at refineries). In addition, they reported that instructors lacked knowledge of regional and local issues associated with cleanup (e.g., local conditions; waterways). On the other hand, Asian (Vietnamese) and Hispanic workers identified issues related to literacy and English as a second language that interfered with training effectiveness and knowledge acquisition. In addition, some workers cited specific design and delivery components that promoted learning including opportunities for hands-on practice during training, small group discussions, use of visuals, and instructors being cognizant of pacing in terms of the presentation of material.

In answer to Research Question 4, “To what extent do disaster cleanup workers from different indigenous, racial-ethnic and immigrant groups transfer knowledge and skill via actions taken (i.e., engaging in safe work behavior) to the cleanup site?”, we found significant differences in overall safety performance. As shown in Table 2, we observed differences in overall safety performance relative to two subsets. That is, all racial/ethnic groups demonstrated significantly higher safety performance in comparison to the Isleños group. The chart in Figure 1, presents mean differences between these racial-ethnic clusters on the overall training safety performance measure. Importantly, as presented in Table 3, the d-statistic for the difference between these groupings was 1.50, indicating that the average performance of the majority cluster (i.e., Asian–Black–Hispanic–Native American–White) was at approximately the 93rd percentile of the Isleños group. As presented in Table 6, this general pattern of racial-ethnic group difference in safety performance was also observed for each dimension of safety performance.

In addressing Research Question 5, “What are the factors that facilitate or hinder training transfer among disaster cleanup workers?”, we identified several themes in workers’ open-ended replies that also clarified why the above subgroup differences in safety performance were found. Notably, many disaster cleanup workers reported that the frequency that they engaged in some safety behaviors was adversely affected by the lack of resources. The reasons provided for the lack of resources were numerous including, for instance, a complete lack of safety equipment and a lack of a particular component such as masks. Related to this, a number of respondents reported that they did not use the equipment due to it being cumbersome or interfering with their work (e.g., gloves stuck to oil).

Workers also reported that, in many cases, safety messaging and related supervisory support for safety was lacking. For instance, a number of workers indicated that expectations were not clear or that the importance of safety was not emphasized. Some of these workers also reported the belief that voicing their concerns for safety would have resulted in some form of reprisal.

**Figure 2:**
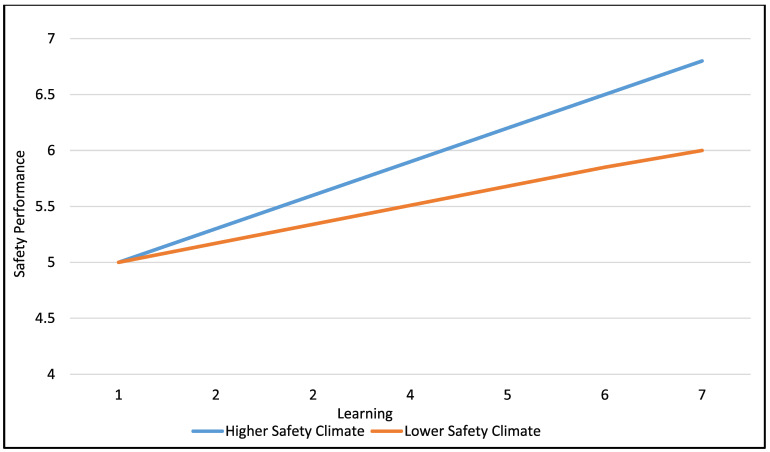
Plot of interaction of safety climate and learning on safety performance.

Results pertaining to the expected interaction of safety climate and learning on safety performance are presented in Table 7. As shown in Table 7, safety climate and learning interacted as expected in influencing overall safety performance. To plot this interaction (see Figure 2), we used a split based on lower (less than 5 on the 7-point scale) versus higher (greater than or equal to 5 on the 7-point scale) values on safety climate. The relationship between learning and overall safety performance was 0.25 when safety climate was lower and 0.56 when safety climate was higher.

In response to Research Question 6, “Do disaster cleanup workers themselves or their co-workers experience any accidents, illnesses, and injuries?”, the vast majority of workers (over 80%) did not report experiencing any accident, injury, or illness due to work. The most common issues that were reported were heat exhaustion, feeling ill from reactions to dispersants, and feeling ill from exposure to fumes due to lack of masks or air respirator usage. Another common category was reporting awareness of a co-worker being ill due to exposure to a hazardous substance. A few respondents cited that accidents, injuries, and illnesses were not reported due to fear of reprisal (job would be taken away).

## 4. Discussion

The primary aim of this case study was to evaluate the training-related experiences of disaster cleanup workers from different racial/ethnic groups in the wake of the Deepwater Horizon Oil Spill. We organized our evaluation with respect to Kirkpatrick’s [16] training evaluation framework (i.e., trainees’ reactions, learning, behavior, and outcomes); cognitive, affective, and skill-based components of learning [19]; and general dimensions of safety performance concerning the use of personal protective equipment, engagement in safe work practices, communication of safety information, and exercise of one’s rights and responsibilities [21]. In doing so, we posed a series of research questions focused on the extent to which workers from six racial/ethnic groups (i.e., Asian (Vietnamese), Black, Hispanic, Isleños, Native American, and White) differentially experienced, learned from, and transferred safety training to cleanup sites. Overall, the results indicated that training was designed and delivered in an effective manner, trainees acquired the necessary knowledge and skills, and trainees transferred their learning insofar as engagement in safe work behavior was concerned. Further, most workers reported that that they were not involved in any accidents or experience any injury or illness from cleanup work. However, when considering the race/ethnicity of the workers, the results also indicated that workers from sub-populations most vulnerable to the effects of the oil spill were not as positively affected by the safety training. Below, we discuss specific racial/ethnic group differences that were observed, and the implications of these findings for future Federal efforts to conduct safety training and promote its transfer for a diverse disaster response workforce.

### 4.1. Effectiveness of Disaster Response Worker Training: Reactions and Learning

Regarding Research Question 1, individuals reported that the design and delivery of safety training was less effective for Isleños and Asian/Vietnamese workers. To a large extent, this pattern of racial/ethnic group differences was also apparent for cognitive, skill, and affective (empowerment) components of learning (Research Question 2). Further, responses from members of these racial/ethnic groups suggest that for safety training to be perceived as effective, it must be viewed as salient and useful for individuals that work and reside in the contaminated region. More specifically, open-ended responses gathered to address Research Question 3 suggest that the training content must emphasize local and regional issues. In addition, for U.S. workers with English as a second language (Vietnamese and some Isleños workers), the training format is particularly important. On suggestion would be to have interpreters in the classroom or deliver training in the primary language of the audience. Another recommendation is to incorporate greater use of hands-on activities into training. These applications should include specific examples relevant to the disaster work they would be engaging in within their communities. Finally, we would recommend that trainers place relatively more emphasis on visuals in comparison to written materials (unless written materials are translated). Together, these suggestions would be expected to positively influence the effectiveness of training for workers with general literacy issues.

Our recommendations in relation to language and hands-on training are particularly relevant for Vietnamese cleanup workers in the U.S. Gulf Coast region. As Carruth et al. [27] found, conducting hands-on training among Vietnamese who reside along the Gulf Coast by experienced fishermen in their language, specifically targeting captains, was considered key to establishing positive safety climates on vessels. Similar results have been found with respect to improving effectiveness of health and safety training initiatives targeting at-risk workers with language and literacy barriers in high-reliability industries in the U.S. and European countries [28,29]. Together, our recommendations are consistent with the literature on alternative means for communicating safety information for racial/ethnic minorities and underserved populations as well the literature on participatory-based safety training methods [9,30,31,32].

### 4.2. Effectiveness of Disaster Response Worker Training: Behavior and Outcomes

Concerning Research Question 4, Isleños cleanup workers generally, in comparison to all other racial/ethnic groups, reported less frequent engagement in all forms of safe work behavior. A notable exception to this observation was that Asian and Hispanic workers, comparable to Isleños workers, also reported less frequent exercising of one’s rights and responsibilities. Another exception was that Asian workers, like Isleños workers, also had somewhat lower use of personal protective equipment in comparison to other racial/ethnic groups.

Open-ended responses gathered to address Research Question 5 indicated that the organizational and managerial aspects of the worksite were important factors influencing when workers would engage in safe work behavior. More specifically, organizational support in relation to providing personal protective equipment and managerial support regarding the transfer of learning were not consistent across work sites. Likewise, the lack of managerial emphasis on safety and fear of reprisal for communicating safety issues were noted as reasons for less frequent safe work behavior. The importance of supervisory support in enhancing successful transfer of the training to working safely also has been demonstrated in studies involving migrant workers in high reliability industries [28,29]. The emphasis on safety, including availability of equipment and encouraging upward communication of safety information, are necessary to ensure that clean-up activities are performed safely on a worksite that is culturally diverse. These findings are consistent with calls for enhanced training in soft skills (communication with supervisors, safety-related values) to enhance transfer of safety training among a culturally diverse workforce [28].

Our observations as to why particular types of safe work behavior were differentially displayed across racial/ethnic groups are consistent with the finding that safety climate positively moderated the relationship between learning and safety performance. More specifically, our measure of safety climate focused on the measurement of supervisory support for the transfer of training and the provision of personal protective equipment, where we found that when these conditions were more positive the relationship between learning and safety performance was greater. As such, the latter result for disaster cleanup workers is consistent with a large body of research on the importance of establishing a work environment that is conducive to the transfer of safety knowledge and skill [22]. Moreover, it is critical that these factors are identified and addressed to promote the maintenance and generalization of the safety training to the worksite [33].

As noted above, regarding Research Question 6, most cleanup workers (over 80%) did not experience any accident, injury, or illness due to work. Notably, when workers experienced a health issue such as heat exhaustion or feeling ill, they were often aware of why the issue occurred. For instance, there was recognition on the behalf of workers that reactions to dispersants and feeling ill from exposure to fumes were due to a lack of use of personal protective equipment (e.g., a mask or air respirator). Likewise, heat exhaustion was recognized as being due to a lack of hydration or the failure to taking rest breaks. Underreporting of accidents, injuries, and illnesses was also cited due to fear of reprisal (job loss). Together, these open-ended responses to questions about the experience of illness point to the need to establish a positive work environment for cleanup workers that supports safety and encourages the continual display of safe work behaviors.

### 4.3. Potential Limitations and Additional Insights

The present case study presents a comprehensive evaluation that provides information regarding the effects of individual and organizational factors influencing the effectiveness and impact of the safety training for a diverse response workforce involved in the cleanup of a large-scale disaster. However, several potential and actual limitations to the study exist. First, our study relied upon a convenience sample of workers, and thus the size and composition of our sample limits generalizability of results to the population of Deepwater Horizon oil spill responders. In addition, the present study utilized self-report measures of training outcomes obtained at one point in time. The concurrent measurement of training outcomes may have contributed in some manner to inflating bivariate relationships between training outcome variables and consistency in findings across training outcomes. It would be beneficial to examine standardized (knowledge tests) and objective measures (health and safety records) along with self-reported measures in future studies. It should be noted, however, that respondents were assured anonymity to reduce bias and engender more honest responses. Third, pre-training assessments were not obtained, which limits our ability to conclude that training explained group differences in knowledge acquisition and the demonstration of safe work behavior. Finally, while the present case study examined transfer of training to clean-up behaviors, a longitudinal study would be needed to gather follow-up measures of health and safety outcomes (injuries, illnesses) over time to measure the longer-term impacts of training.

Also worth noting is the active support and participation of the local community-based and faith-based organizations in the evaluation process. The evaluation team partnered with these organizations to assist with the recruitment of participants and survey administration. The long-standing ties in the community and trusting relationships associated with these organizations were instrumental in engaging participants in the evaluation process, in particular for gaining access to the Vietnamese and Isleños workers. Community partnerships have been recognized as integral to the safety training process for limited or non-English speaking workers in other disaster recovery efforts, such as those involved in the World Trade Center cleanup [34]. Public health agencies and researchers have prioritized the development of partnership with community-based organizations to reach specific worker populations [35]. Therefore, inclusion of these partners early in the planning stages of the training and evaluation process is recommended.

Furthermore, the present study focused solely on traditional, classroom-based delivery of the safety training. Recently, the COVID-19 pandemic has caused a rapid transition from traditional face-to-face delivery of occupational health and safety training to the use of distance learning methods. Preliminary evidence indicates that use of synchronous distance learning methods for peer-based participatory worker safety training results in similar safety knowledge gains and transfer compared to that delivered in traditional face-to-face format [36]. Because distance learning (online) safety training is a time- and cost-efficient method, its use should be explored for training disaster workers responding to large scale natural and man-made disasters. However, additional evidence is needed with respect to evaluating the effectiveness and impact of such training across worker subpopulations, particularly those who are non-English speaking and have low educational levels.

An additional area of inquiry for the disaster response workforce is related to the impact of the disaster on the mental and physical aspects of the workers. While the present training evaluation focused largely on behavioral issues, the qualitative responses suggested that psychological factors were also important. Thus, consideration could be given to interventions that synergistically address the mental and behavioral issues that occur, particularly for those who reside in the communities that are affected and are an active part of the clean-up activities. For example, worker resiliency training would be a potentially useful complement to the safety training that addresses both the physical and mental capacity of the worker in successful response and recovery efforts.

Finally, we stress that the evaluation framework and related methods and measures used in the current study can be adapted with minor modifications to assist in the evaluation of safety and health training efforts for other emergency response and recovery interventions across various regions and types of emergencies/disasters. In this way, the present systematic approach to safety training evaluation not only offers information for structuring a training evaluation effort, but also provides useful information concerning the attitudinal, learning, and behavioral elements that should be considered in the design and delivery of training for disaster cleanup workers.

## 5. Conclusions

Our evaluation of health and safety training in the wake of the Deepwater Horizon oil spill points to the need to consider such training in a broad context. That is, where training professionals consider the entire training system (i.e., training needs, delivery method, etc.) as well as safety climate for the use of training to ensure training effectiveness and its transfer across worker subpopulations. In this regard, our evaluation effort illustrates how a recognized training evaluation framework, aspects of learning, and general dimensions of safety performance can be employed to systematically evaluate response worker training for natural and man-made disasters and provide information for modifications as needed for a culturally diverse workforce, including those most vulnerable to risks associated with disaster response work. Collectively, the results from applying this systematic approach can be used as part of efforts to ensure continuous quality improvement in safety training and mitigation responses aimed at both handling the cleanup and maintaining the health and safety of a diverse disaster response workforce.

## Figures and Tables

**Table 1:** Descriptive statistics and correlations between composite study variables.

Variable	Mean	SD	1	2	3	4	5	6	7
1. Reactions to Training	5.35	1.82	(0.98)						
2. Learning	5.35	1.66	0.81	(0.95)					
3. Using Personal Protective Equipment	6.02	1.28	0.60	0.70	(0.93)				
4. Engaging in Work Practices to Reduce Risks	6.15	1.16	0.63	0.71	0.87	(0.95)			
5. Communicating Safety Information	5.90	1.40	0.64	0.64	0.69	0.79	(0.97)		
6. Exercising Employee Rights and Responsibilities	6.19	1.43	0.48	0.47	0.48	0.59	0.77	(0.94)	
7. Safety Performance (Overall)	6.03	1.19	0.63	0.63	0.86	0.93	0.84	0.80	(0.98)

Note. SD = standard deviation. Pairwise Ns ranged from 272 to 456. All correlations are statistically significant at *p* < 0.01. Internal consistency reliability (Cronbach’s alpha) estimates are reported along the diagonal.

**Table 2:** ANOVA results for overall study measures with post-hoc Tukey tests for racial-ethnic group differences.

	Racial/Ethnic Group													
	Native American		White		Black		Hispanic		Asian		Isleños		F (df)	*η* ^2^
Measure	M	SD	M	SD	M	SD	M	SD	M	SD	M	SD		
Reactions to Training	5.22 ^2,3^	1.54	6.30 ^3^	1.23	6.08 ^3^	1.54	5.76 ^3^	1.75	4.02 ^1,2^	1.86	3.81 ^1^	0.78	41.53 ** (5, 413)	0.32
Learning	4.58 ^1^	2.23	6.11 ^3^	1.48	5.96 ^2,3^	1.81	5.83 ^2,3^	1.83	4.94 ^1,2^	1.32	4.05 ^1^	0.61	27.31 ** (5, 447)	0.23
Safety Performance	6.91 ^2^	0.18	6.65 ^2^	0.91	6.71 ^2^	0.93	6.23 ^2^	1.15	5.89 ^2^	0.66	4.60 ^1^	0.43	59.59 ** (5, 325)	0.48

Note: M = mean, SD = standard deviation. df = degrees of freedom. For post-hoc Tukey HSD tests, mean values with a superscript of 1, 2, and 3 represent homogeneous subsets (i.e., clusters of groups) that are different from other subsets, with a subset value of 1 being lower than a subset value of 2 and a subset value for 3 being greater than a subset value of 2. A group mean associated with two subsets is not significantly different from other group means within the respective subsets. ** *p* < 0.01.

**Table 3:** Standardized differences between racial-ethnic subsets on overall reactions to training, learning, and safety performance.

Variable: Racial-Ethnic Subset 1/ Racial Ethnic Subset 2	Cohen’s d (95% c.i.)
Reactions to Training: Black-Hispanic-Native American-White/ Asian-Isleños	1.40 (1.18, 1.62)
Learning: Black-Hispanic-White/ Asian-Isleños-Native American	1.01 (0.81, 1.21)
Safety Performance: Asian-Black-Hispanic-Native American-White/ Isleños	1.50 (1.25, 1.76)

Note. c.i. = 95% confidence interval. Subset comparisons are only made for mutually exclusive subsets identified in Table 2 for the respective composite measures. For Reactions and Learning, mutually exclusive subsets were formed by combining groups from subsets 1 and 3, respectively, as specified in Table 2.

**Table 4:** ANOVA results for specific assessments of training (reactions) with post-hoc Tukey tests for racial-ethnic group differences.

	Racial/Ethnic Group													
	Native American		White		Black		Hispanic		Asian		Isleños		F (df)	*η* ^2^
Training Assessment	M	SD	M	SD	M	SD	M	SD	M	SD	M	SD		
Presentation of training according to the needs of the trainees.	5.56 ^3^	1.42	6.31 ^3^	1.25	6.09 ^3^	1.61	5.55 ^3^	1.94	4.06 ^2^	1.89	2.83 ^1^	1.09	76.70 (5, 479)	0.45
Usefulness and ease of understanding materials and handouts.	4.89 ^1,2^	2.26	6.28 ^3^	1.30	6.08 ^3^	1.56	5.97 ^2,3^	1.59	3.98 ^1^	1.97	3.76 ^1^		37.29 (5, 423)	0.31
Adequacy of training for preparing oneself for Gulf Oil Spill cleanup activities.	5.11 ^2,3^	2.09	6.05 ^3^	1.57	5.93 ^3^	1.85	6.10 ^3^	1.34	4.67 ^1,2^	1.44	1.79 ^1^	1.12	103.28 (5, 472)	0.52

Note: M = mean, SD = standard deviation. df = degrees of freedom. For post-hoc Tukey HSD tests, mean values with a superscript of 1, 2, and 3 represent homogeneous subsets (i.e., clusters of groups) that are different from other subsets, with a subset value of 1 being lower than a subset value of 2 and a subset value for 3 being greater than a subset value of 2. A group mean associated with two subsets is not significantly different from other group means within the respective subsets.

**Table 5:** ANOVA results for learning components with post-hoc Tukey tests for racial-ethnic group differences.

	Racial/Ethnic Group													
	Native American		White		Black		Hispanic		Asian		Isleños		F (df)	*η* ^2^
Learning Component	M	SD	M	SD	M	SD	M	SD	M	SD	M	SD		
Cognitive (Declarative Knowledge)	4.33 ^1^	2.06	6.11 ^3^	1.56	5.84 ^2,3^	1.95	5.63 ^2,3^	2.06	4.87 ^1,2^	1.45	3.85 ^1^	1.01	25.68 ** (5, 459)	0.22
Skill (Procedural Knowledge)	4.67 ^1^	2.12	5.97 ^3^	1.79	5.89 ^2,3^	1.90	6.03 ^2,3^	1.79	4.81 ^1,2^	1.48	4.31 ^1^	0.70	18.02 ** (5, 465)	0.16
Affective (Empowerment)	4.75 ^1,2^	2.32	6.08 ^4^	1.54	5.89 ^3,4^	1.90	5.83 ^2,3,4^	1.78	4.92 ^1,2,3^	1.51	3.94 ^1^	0.73	27.03 ** (5, 459)	0.23

Note: M = mean, SD = standard deviation. df = degrees of freedom. For post-hoc Tukey HSD tests, mean values with superscripts of 1, 2, 3, and 4 represent homogeneous subsets (i.e., clusters of groups) that are different from other subsets, with a subset value of 1 being the lowest and successive, higher subset numbers being greater than the lower subsets. A group mean associated with multiple subsets is not significantly different from other group means within the respective subsets. ** *p* < 0.01.

**Table 6:** ANOVA results for safety performance dimensions with post-hoc Tukey tests for racial-ethnic group differences.

	Racial/Ethnic Group													
	Native American		White		Black		Hispanic		Asian		Isleños		F (df)	*η* ^2^
Safety Performance Dimension	M	SD	M	SD	M	SD	M	SD	M	SD	M	SD		
Using Personal Protective Equipment	6.87 ^3^	0.24	6.52 ^2,3^	1.30	6.61 ^3^	1.10	6.32 ^2,3^	1.38	5.67 ^1,2^	1.09	4.81 ^1^	0.41	37.80 ** (5, 390)	0.33
Engaging in Work Practices to Reduce Risk	6.50 ^2,3^	0.47	6.66 ^2,3^	0.92	6.73 ^3^	0.99	6.43 ^2,3^	1.01	5.97 ^2^	0.69	4.61 ^1^	0.47	65.65 ** (5, 355)	0.48
Communicating Safety Information	7.00 ^3^	0.00	6.59 ^3^	1.17	6.63 ^2,3^	1.12	5.72 ^2^	1.58	5.92 ^2,3^	0.70	4.29 ^1^	0.76	64.19 ** (5, 359)	0.45
Exercising Employee Rights and Responsibilities	6.78 ^2^	0.38	6.53 ^2^	1.31	6.52 ^2^	1.21	5.34 ^1,2^	2.26	5.74 ^1,2^	0.71	4.63 ^1^	2.00	8.73 ** (5, 283)	0.13

Note: M = mean, SD = standard deviation. df = degrees of freedom. For post-hoc Tukey HSD tests, mean values with superscripts of 1, 2, and 3 represent homogeneous subsets (i.e., clusters of groups) that are different from other subsets, with a subset value of 1 being the lowest and successive, higher subset numbers being greater than the lower subsets. A group mean associated with more than one subset is not significantly different from other group means within the respective subsets. ** *p* < 0.01.

**Table 7:** Results of regression for the prediction of overall safety performance from safety climate perceptions and learning.

Safety Performance			
Model	Predictor Variable	β	β
1	Safety Climate	0.52 **	
	Learning	0.19 **	
2	Safety Climate		0.61 **
	Learning		0.32 **
	Safety Climate X Learning		0.34 **
	R^2^	0.47 **	0.54 **
	R^2^		0.07 **

Note. N = 239. ** *p* < 0.01.

## Data Availability

The data presented in this study may be requested from the senior author. The data are not publicly available due to this project being a Federal demonstration project.

## Appendix A

Survey Items and Dimensions

**Reaction Items:**

The training was presented effectively according to needs of the trainees (e.g., language, culture, educational level).

The materials and handouts were useful and easy to understand.

I felt that the training adequately prepared me for the Gulf Oil Spill clean-up activities.

**Learning Items:**

I was knowledgeable about potential hazard and mitigation strategy associated with the

Gulf Oil activities I performed.

I was knowledgeable about how to minimize potential exposure to hazards associated with the Gulf Oil Spill activities to myself, my co-workers, and the public.

I was empowered to identify and avoid hazards while performing the Gulf Oil Spill activities.

**Behavior (Safety Performance) Items and Dimensions:**

**Using Personal Protective Equipment**

I used the appropriate personal protective equipment (for example: sunscreen, gloves, Tyvek, goggles, boots) as indicated by the site health and safety plan.

I put on all personal protective equipment correctly.

I removed all personal protective equipment correctly.

When required, I properly assisted partner (buddy system) in putting on and removing personal protective equipment.

I properly performed clean-up work while wearing personal protective equipment.

**Engaging in Work Practices to Reduce Risk**

I correctly used applicable hazard controls and equipment (for example: rake, shovel, pulley, hook).

I applied the appropriate work practices to reduce exposures to hazards (for example: work/rest cycle, hydration, use of equipment to pick up product) relating to operations and environmental clean-up.

I properly disposed of materials and/or equipment that pose a health risk (for example: contaminated equipment, contaminated clothing).

I practiced safe spill handling procedures (for example: using a shovel to pick up tarballs; using glove to touch oil covered boom; putting oil covered material in plastic bags).

I took action to avoid contamination (for example: not stepping in product; eating/drinking away from work areas; washing hands; not wearing work clothes off-site).

I accurately followed established decontamination procedures (for example: cleaning up before you leave the worksite; properly taking off and disposing of Tyvek).

I took appropriate action (for example: drinking more water, staying in the shade, wearing loose fitting, wearing light-colored clothes) to prevent recurrence of injuries, illnesses, accidents, and/or near misses (for example: heat-related illnesses, pinches, puncture wounds, sun exposure).

**Communicating Health and Safety Information**

When necessary, I communicated potential exposures (for example: oil on hands, oil splashed on face) to key personnel (boat captain, team leader, supervisor) responsible for site health and safety.

I contacted appropriate personnel (boat captain, team leader, supervisor, site safety officer) when faced with questions and/or issues regarding environmental clean-up.

I reported incidents, accidents, and/or illnesses (health and safety officer, front-line supervisor).

I engaged in the appropriate methods to notify workers, supervisors, and/or emergency coordinators of emergency conditions.

**Exercising Employee Rights and Responsibilities**

I appropriately used reference materials that may provide additional health and safety information (for example: Safety and Health Awareness for Oil Spill Clean-up Workers pocket guide; MSDS).

When necessary, I exerted the employee’s rights to provide input into altering the site health and safety plan. For example, if you saw something dangerous, you brought it to your supervisor’s attention.

When prevented from or punished for exercising my rights under OSHA policies and procedures, I took the appropriate steps. For example: reporting an unsafe situation to another person because it didn’t get fixed; reporting if you were threatened because you said something was unsafe

**Safety Climate Items:**

My supervisor on-site supported the training I received.

The appropriate personal protective equipment was provided.
